# A prognostic signature derived from ac4C-associated genes stratifies survival and tumor immune microenvironment in cutaneous melanoma

**DOI:** 10.3389/fimmu.2025.1727135

**Published:** 2026-01-07

**Authors:** Qiaoying Jin, Na Jiang, Guoxiu Chen, Jiarui Zhu, Haiyu Niu, Yiheng Han, Chao Li, Shixiong Wang, Yali Liu

**Affiliations:** 1 Cuiying Biomedical Research Center, The Second Hospital & Clinical Medical School, Lanzhou University, Lanzhou, China; 2 Department of Oncology, The Second Hospital & Clinical Medical School, Lanzhou University, Lanzhou, China; 3 Department of Cardiac Surgery, Shanghai Fourth People’s Hospital, Shanghai, China

**Keywords:** epitranscriptomics, immune microenvironment, machine learning, N4-acetylcytidine (ac4C), prognostic signature, skin cutaneous melanoma

## Abstract

The clinical management of cutaneous melanoma (SKCM) is particularly challenging due to the disease’s high degree of heterogeneity, which leads to unpredictable patient outcomes and highly variable responses to therapy. Clinicians are in urgent need of a reliable molecular profile system to predict patient trajectory and guide therapeutic decisions. Recent research emphasizes the pivotal role of N4-acetylcytidine (ac4C) modification, a novel epigenetic mechanism, in promoting diverse human diseases pathogenesis and progression. Nevertheless, its specific impact and the clinical relevance of ac4C-associated genes in SKCM remain to be elucidated. This study aimed to develop an “ac4C-associated Gene Signature” (AGS) to stratify patient prognosis, inform therapeutic decisions, and advance biological insight into cutaneous melanoma. Through integrative analysis of ac4C-related genes in SKCM, we identified 41 differentially expressed candidates and derived three molecular subtypes with distinct clinical outcomes. We subsequently constructed a stable seven-gene signature using Cox-LASSO regression, which effectively stratified patients into high- and low-risk groups in the TCGA cohort and was validated in an independent GEO dataset. The AGS not only predicted survival but also characterized the tumor-immune microenvironment, distinguishing immunologically “hot” from “cold” phenotypes, and suggested potential responses to immunotherapy and chemotherapy. Additional support from the HPA database, cell line models, and RT−qPCR experiments supported the model’s biological relevance. In summary, this study provides a clinically applicable prognostic tool for risk stratification and personalized treatment planning in SKCM.

## 1 Introduction

Cutaneous malignant melanoma (SKCM) accounts for approximately 5% of all cancer diagnoses. Its high malignancy and strong metastatic potential make it a significant threat to human health. The clinical management of SKCM remains challenging, as patient responses to surgery, targeted therapy, and immunotherapy are often unpredictable. A core difficulty lies in the lack of reliable biomarkers for predicting therapeutic efficacy and patient prognosis. Therefore, establishing a robust biomarker system for risk stratification, treatment response prediction, and toxicity assessment is critical for improving SKCM outcomes (1).

In recent years, epitranscriptomics has emerged as a promising field for uncovering novel regulatory mechanisms in cancer. This discipline examines how chemical modifications to RNA govern its function, enabling dynamic and reversible regulation of gene expression without altering the underlying nucleotide sequence - a form of “epigenetics at the RNA level” (2). To date, over 170 distinct RNA modifications have been characterized. Among these, N6-methyladenosine (m6A) is the most extensively studied, regulating key mRNA processes like splicing, stability, and translation, and is implicated in all stages of tumorigenesis (3–5). This has spurred growing interest in other modifications, including N4-acetylcytidine (ac4C), pseudouridine (Ψ), 5-methylcytidine (m5C), and N1-methyladenosine (m1A) (6, 7).

These modifications can alter RNA structure, mediate RNA-protein interactions, or recruit effector molecules, collectively forming a complex “RNA modification regulatory network” with pro- or anti-tumor roles in various cancers. Advances in this field have not only deepened our understanding of tumor mechanisms but also provided new opportunities for developing biomarkers and targeted therapies. For example, inhibitors targeting m6A regulatory proteins (e.g., “writers” and “erasers”) have entered early-stage drug development (8), underscoring the translational potential of epitranscriptomics in precision oncology.

Ac4C, catalyzed primarily by the acetyltransferase NAT10, serves as a critical epitranscriptomic modulator. It influences RNA fate by enhancing mRNA structural stability, augmenting translation efficiency, and ensuring translational fidelity (5, 9, 10). Mechanistically, ac4C alters the charge distribution and secondary structure of RNA, protecting mRNAs from nuclease-mediated degradation and facilitating ribosome binding, thereby increasing protein yield and accuracy (11). In tRNA, ac4C modification within the anticodon loop helps maintain codon recognition accuracy (12). Through these roles, ac4C establishes a crucial link between genetic information and functional proteome dynamics.

Emerging evidence has established diverse oncogenic functions for ac4C across cancer types. It enhances glycolysis and lactate secretion in tumor cells, shaping an immunosuppressive microenvironment that amplifies regulatory T cell (Treg) function and facilitates immune escape (13). In bladder carcinoma, NAT10-mediated ac4C deposition on AHNAK transcripts enhances mRNA stability, amplifies the DNA damage response, and promotes platinum-based chemotherapy resistance (14). In triple-negative breast cancer, ac4C stabilizes JunB mRNA, driving aerobic glycolysis and immunosuppression, suggesting that its pharmacological inhibition could augment immunotherapy response (15). Additional roles include regulating translation efficiency in esophageal cancer (16), conferring capecitabine resistance in breast cancer (17), enhancing treatment tolerance through checkpoint activation (18, 19), and synergizing with m6A reading proteins to modulate glycolysis in osteosarcoma (20). Ac4C also stabilizes non-coding RNAs in nasopharyngeal carcinoma (21), promotes glycolytic addiction in gastric cancer (22), and influences the progression of hematological malignancies (23). However, the overall functional patterns and clinical significance of ac4C modification in SKCM remain largely unexplored.

Therefore, we initiated a systematic investigation of ac4C-associated genes in SKCM. This study aims to: first, establish a molecular classification based on ac4C-associated gene expression patterns; and second, develop a quantifiable risk assessment system - the ac4C-associated Gene Signature (AGS) - to predict patient survival, characterize the tumor immune microenvironment, and suggest potential therapeutic associations.

We anticipate that the AGS will hold substantial potential for clinical translation. By integrating diverse data types with machine learning algorithms, this system may enable accurate prognosis discrimination and personalized treatment guidance. Furthermore, elucidating the role of ac4C-associated genes in SKCM could provide a foundation for novel therapies targeting RNA-modifying enzymes. These insights are expected to contribute to the advancement of precision oncology in SKCM, thereby potentially enhancing therapeutic efficacy and long-term survival for affected individuals.

## 2 Materials and methods

### 2.1 Data acquisition

Data Transcriptomic and clinical data were obtained from the Xena database, comprising 472 skin cutaneous melanoma (SKCM) samples and 812 normal controls. Specimens lacking complete survival information were excluded from subsequent analysis. For independent validation, an external validation cohort was assembled, including 214 samples from the GSE65904 dataset, as well as 46 cutaneous melanoma samples and 16 normal controls from the GSE15605 dataset (available at https://www.ncbi.nlm.nih.gov/geo/).

### 2.2 Detection and screening of ac4C-associated differentially expressed genes

A comprehensive set of 2,156 ac4C-associated genes was compiled through a systematic literature review (24). This collection encompasses two categories: (1) known regulatory proteins of ac4C modification (e.g., the “writer” NAT10); and (2) genes whose mRNAs have been experimentally validated as direct functional targets of ac4C modification in prior studies (e.g., identified through ac4C-seq or RIP-qPCR in references (5, 9, 10, 13–15)). Functional enrichment analysis confirmed that this gene set is significantly associated with biological processes central to ac4C function, including mRNA metabolism and translation (**Supplementary Figure 3**), thereby supporting its biological coherence as a foundation for this study. Differential expression profiling between SKCM tumors and normal skin samples from the TCGA cohort was then applied to this gene set to identify which ac4C-associated genes were significantly dysregulated. Protein-protein interaction data for the resulting differentially expressed genes were obtained from the STRING database (https://cn.string-db.org).

### 2.3 Consistent clustering analysis

Consensus clustering was applied to 472 TCGA-SKCM samples using the CNS platform (https://cnsknowall.com) to explore associations between ac4C-related differentially expressed genes and molecular subtypes in cutaneous melanoma. The cluster count parameter k was evaluated across a range of 2 to 10. To visualize expression patterns across subtypes, we generated heatmaps using the CNS package and evaluated survival differences between clusters via Kaplan–Meier analysis. A Gene Signature Associated with ac4C for Prognostic Prediction in SKCM.

### 2.4 An ac4C-based prognostic signature: construction and independent validation

To develop a transcriptome-based prognostic index for cutaneous melanoma, ac4C-associated genes were initially screened by univariate Cox regression (P < 0.05) in the TCGA-SKCM cohort (n = 468). A refined gene set was subsequently derived through LASSO-penalized Cox regression with 10-fold cross-validation (glmnet R package; minimum λ criteria) to mitigate overfitting while eliminating redundant features. This process yielded a refined seven-gene signature, each gene assigned a non-zero regression coefficient (β_1_–β_7_). A risk score for each patient was calculated using the following formula: RiskScore = Σ_i=1_^7^ (β_i_ × E_i_), where E_i_ represents the normalized expression level of gene i. Employing the median risk score from the TCGA cohort as the cutoff, patients were categorized into high- and low-risk subgroups. Kaplan-Meier analysis with log-rank testing demonstrated significant survival disparity between these groups. Principal component analysis (PCA) confirmed distinct transcriptomic profiles separating the two risk categories. The model’s prognostic performance was further quantified by time-dependent ROC analysis, showing predictive accuracy at 1-, 3-, and 5-year intervals. To validate the model, we applied the same coefficients and median cutoff value to an independent validation cohort, GSE65904 (n = 214), which successfully reproduced the risk stratification and allowed for external validation of the prognostic model.

### 2.5 Clinical validation of the prognostic risk model

To determine whether the derived risk signature retains prognostic value in the presence of conventional covariates, we extracted age, sex, TNM stage and histological grade from the TCGA-SKCM data matrix and subjected these variables, together with the continuous risk score, to univariate and multivariable Cox modelling.

### 2.6 Analysis of tumor immune microenvironment

The tumor immune landscape was characterized by profiling immune cell composition, checkpoint molecule expression, and immunophenotypic scores. Abundances of 28 immune cell subsets in skin cutaneous melanoma were quantified using the ssGSEA algorithm implemented on the CloudBioSense platform (http://www.biocloudservice.com/). The resulting infiltration profiles were visualized as a heatmap. The IOBR R package was employed to investigate alterations in immune infiltration profiles and functional activity across distinct risk strata. Furthermore, checkpoint molecule expression was systematically compared to elucidate potential implications for immunotherapeutic responsiveness.

The immunogenicity of tumors and their predicted response to immune checkpoint blockade were inferred using the Immunophenoscore (IPS). This composite metric, curated by The Cancer Immunome Atlas (TCIA; https://tcia.at/), was utilized to characterize the immunologic landscape of the tumor microenvironment.

### 2.7 Chemosensitivity analysis

To investigate the correlation between ac4C-modulated transcripts and chemosensitivity, we selected ten chemotherapeutic agents commonly used in clinical practice. Drug sensitivity, estimated by IC_50_ values through the pRRophetic R package, was compared across risk stratifications.

### 2.8 Experimental validation of the AGS model using CCLE and HPA databases and RT-qPCR analysis

To validate our in-silico predictions with experimental evidence, we stratified melanoma cell line transcriptomes from the Cancer Cell Line Encyclopedia (CCLE) according to the prognostic risk score. Protein-level expression of candidate biomarkers was then examined using immunohistochemistry images from the Human Protein Atlas (HPA), providing histopathological support for their biological relevance.

To experimentally validate gene expression in clinical samples, RT−qPCR was conducted on three SKCM tissues alongside matched adjacent normal tissues. Following TRIzol extraction, RNA quantity and purity were verified on a NanoPhotometer N50 (Implen). Reverse transcription was carried out with the SweScript RT Kit (Servicebio, CN), and quantitative PCR was run in 96-well plates with 2× Universal Blue SYBR Green Master Mix (Servicebio, CN) on a CFX96 system (Bio-Rad). Transcript abundance was normalised to GAPDH and expressed as fold-change calculated by the 2^−ΔΔCt^ algorithm; oligonucleotide sequences are supplied in **Supplementary Table 2** (Tsingke, Beijing, China).

## 3 Results

**Figure 1:**
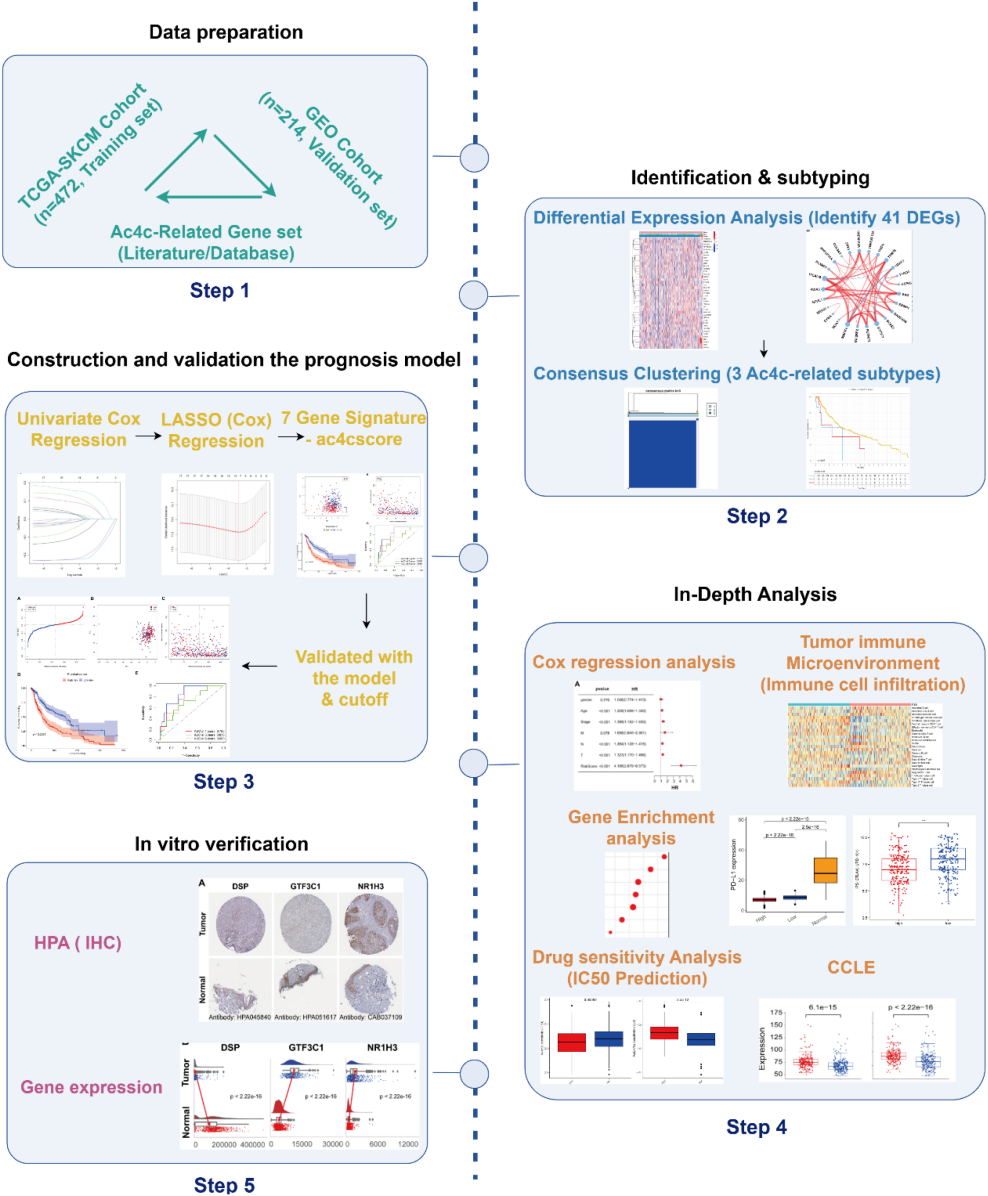
Analytical framework for evaluating ac4C-associated genes in SKCM. The results of this study enhance the current comprehension of ac4C’s functional implications in cutaneous melanoma and offer valuable perspectives for the development of new prognostic biomarkers as well as therapeutic strategies aimed at the ac4C-related pathway.

This study elucidates the functional roles of ac4C-related genes in cutaneous melanoma (**Figure 1**), emphasizing their clinical significance as prognostic biomarkers and potential therapeutic targets. We began by performing a comprehensive profiling of the ac4C-associated gene expression landscape, which identified three distinct molecular subtypes. Subsequently, we developed a seven-gene prognostic signature capable of gauging individual patient risk.

### 3.1 Identification and characterization of 41 ac4C-associated regulatory genes

**Figure 2:**
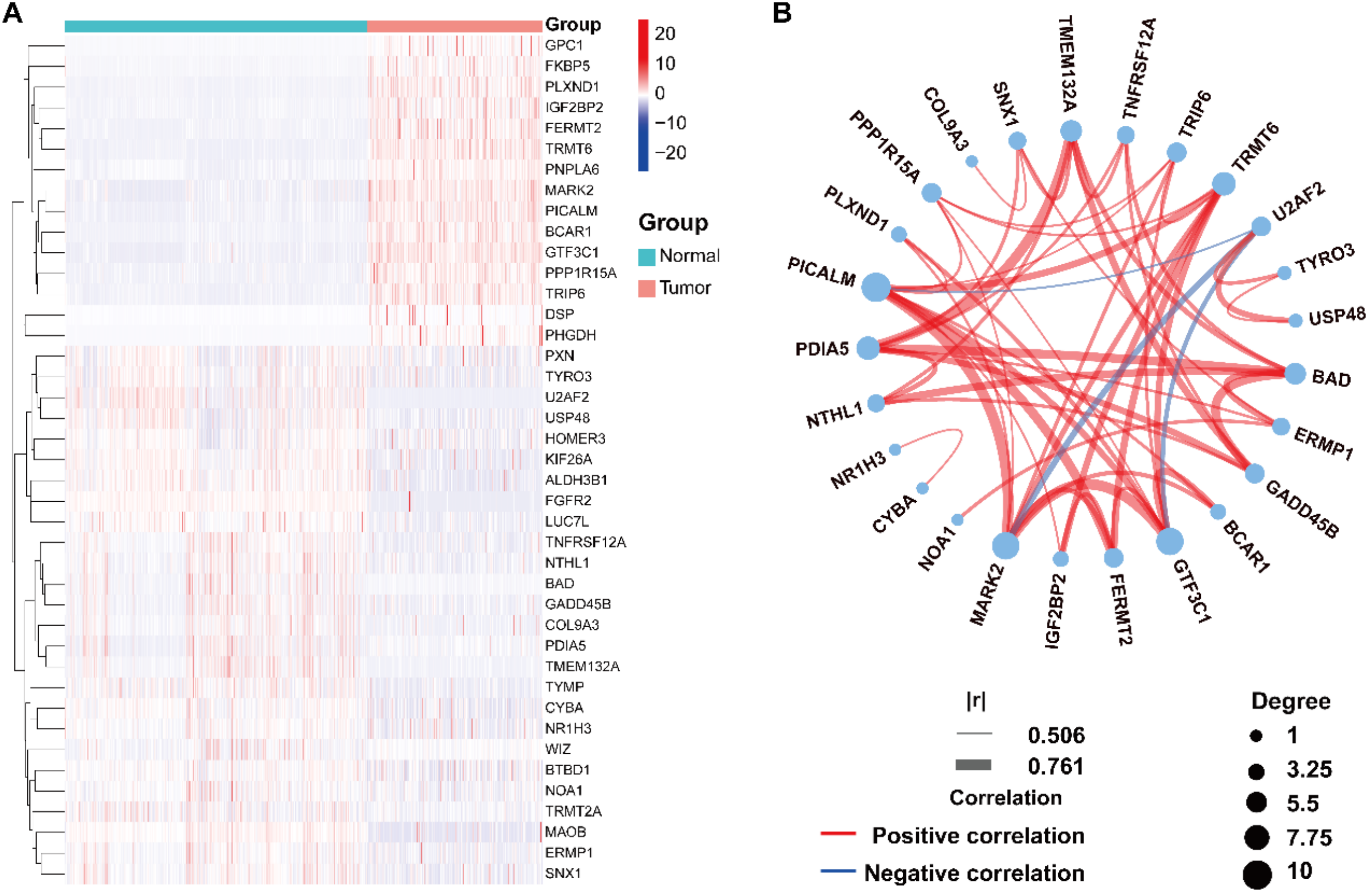
Expression patterns and interaction networks of 41 ac4C-associated genes. **(A)** Volcano plot displaying differential gene expression between normal and tumor tissues (blue: downregulated; red: upregulated). **(B)** Correlation network of ac4C-related genes, with red edges denoting positive interactions and blue edges negative ones; color saturation represents correlation strength.

Transcriptomic profiles from 472 SKCM tumor specimens and 812 normal skin samples were acquired via the Xena database. Analysis of 2,156 ac4C-associated genes for differential expression between tumor and normal tissues identified screen out 41 ac4C key molecules with abnormal behavior in SKCM (|log_2_FC| > 1, p < 0.001), with 15 upregulated and 26 downregulated in tumors (**Supplementary Table 1**). A heatmap was created to display expression profiles, using blue for low expression and red for high expression (**Figure 2A**). Protein–protein interaction was constructed to investigate functional connections from the list of genes. A correlation network diagram further illustrated interconnectivity among the 41 DEGs (**Figure 2B**), with red edges denoting positive correlations. The analysis identified 25 highly connected hub genes, implicating them as key players in SKCM pathogenesis.

### 3.2 Identification of three ac4C-associated molecular subtypes with divergent clinical outcomes

**Figure 3:**
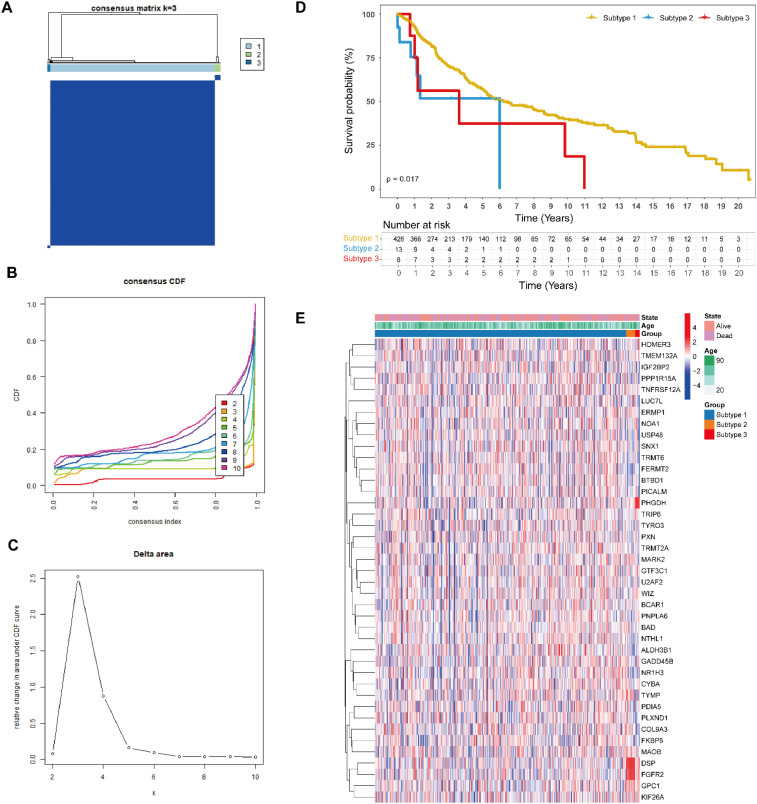
Molecular subtyping of SKCM based on ac4C-associated differentially expressed genes (DEGs). **(A)** Identification of three molecular subtypes through unsupervised consensus clustering. **(B)** CDF illustrating consensus levels for different k values. **(C)** Delta area plot showing changes in clustering stability with increasing (k) **(D)** Kaplan-Meier survival analysis comparing overall survival among the three subtypes. **(E)** Heatmap visualizing expression patterns of ac4C-related DEGs and clinicopathological features across the three subtypes.

To explore the biological and clinical implications of the 41 ac4C-related differentially expressed genes (DEGs) in cutaneous melanoma, we performed consensus clustering on 472 TCGA patients with complete clinical and survival information. Cluster stability was evaluated for k values ranging from 2 to 10, and consensus cumulative distribution function (CDF) analysis indicated that three clusters (k = 3) provided the optimal partitioning and tracking plot analyses (**Figures 3A–C**). These genes, exhibiting distinct molecular ‘faces’, enabled the stratification of seemingly homogeneous SKCM patients into three clinically distinct subgroups. Significant disparities in overall survival were observed between the subtypes using Kaplan-Meier analysis (p = 0.017, **Figure 3D**), underscoring the prognostic value of this classification. A combined heatmap was generated to visualize both gene expression and key clinical variables such as age and survival status (**Figure 3E**); however, most genes exhibited minimal variation in clinical profiles across subtypes.

### 3.3 Construction of the ac4C-associated prognostic signature

**Figure 4:**
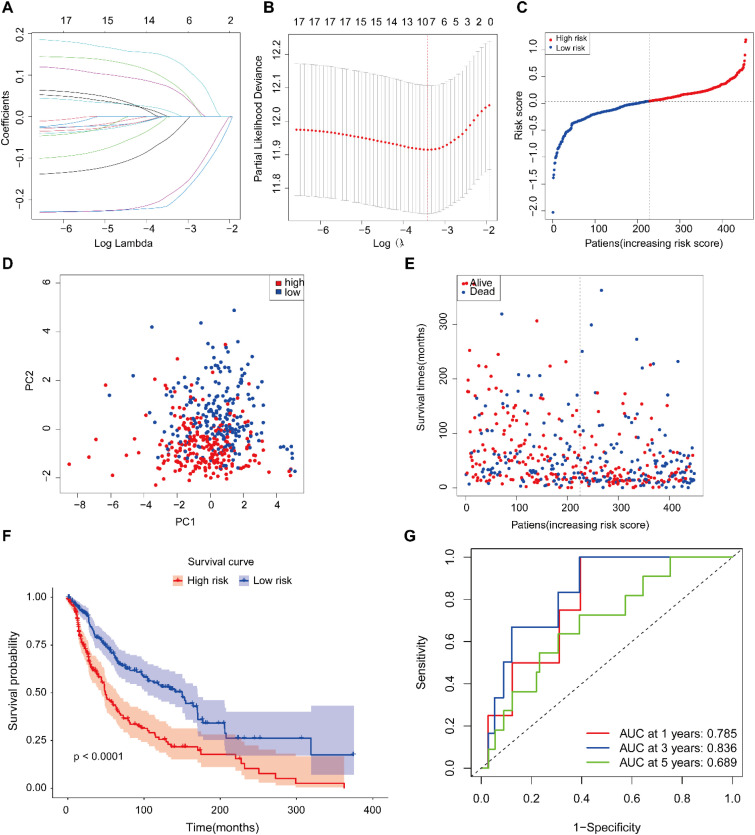
Construction of an ac4C-based prognostic signature in the TCGA-SKCM cohort. **(A)** LASSO regression analysis derived from univariate Cox results of ac4C-related genes. **(B)** Ten-fold cross-validation for determining the optimal penalty parameter in LASSO. **(C)** Distribution of patient risk scores. **(D)** Principal component analysis (PCA) projection of samples stratified by risk score. **(E)** Survival status distribution of patients (dotted line separates low- and high-risk groups). **(F)** Kaplan-Meier survival curves comparing high- and low-risk patients. **(G)** Time-dependent ROC curves evaluating the predictive accuracy of the risk signature.

Utilizing clinical and transcriptomic data from 472 SKCM patients in TCGA, we conducted univariate Cox regression on 41 ac4C-related DEGs, identifying those significantly linked to prognosis (p < 0.001). LASSO regression was then applied to refine the feature set, yielding seven robust prognostic genes: DSP, NR1H3, SNX1, TMEM132A, GTF3C1, NTHL1, and WIZ (**Figures 4A, B**). The AGS, a multivariate prognostic signature, was developed using the formula: Risk score = (0.083356004 × DSP) + (0.069381787 × NR1H3) + (-0.186326629 × SNX1) + (-0.046685347 × TMEM132A) + (0.069381787 × GTF3C1) + (0.011591433 × NTHL1) + (0.140910003 × WIZ). Using the median risk score as cutoff, patients were categorized into high- and low-risk categories (**Figure 4C**). Principal component analysis (PCA) demonstrated a distinct separation between the two risk subgroups (**Figure 4D**). The prognostic signature clearly stratified patients into two groups with divergent clinical outcomes: those in the high-risk group had significantly poorer overall survival, while the low-risk group exhibited a more favorable prognosis (p < 0.001, **Figures 4E, F**). The prognostic signature exhibited good predictive performance for 1-, 3-, and 5-year survival (AUC = 0.785, 0.736, and 0.689, respectively), as determined by time-dependent ROC analysis. The model showed particularly robust discriminatory power for intermediate-term outcomes (**Figure 4G**). In summary, the AGS, constructed from seven ac4C-associated genes, effectively classifies SKCM patients into risk groups with distinct clinical outcomes, offering a potential framework for prognostic assessment and individualized therapeutic strategies.

### 3.4 Independent validation of the prognostic signature

**Figure 5:**
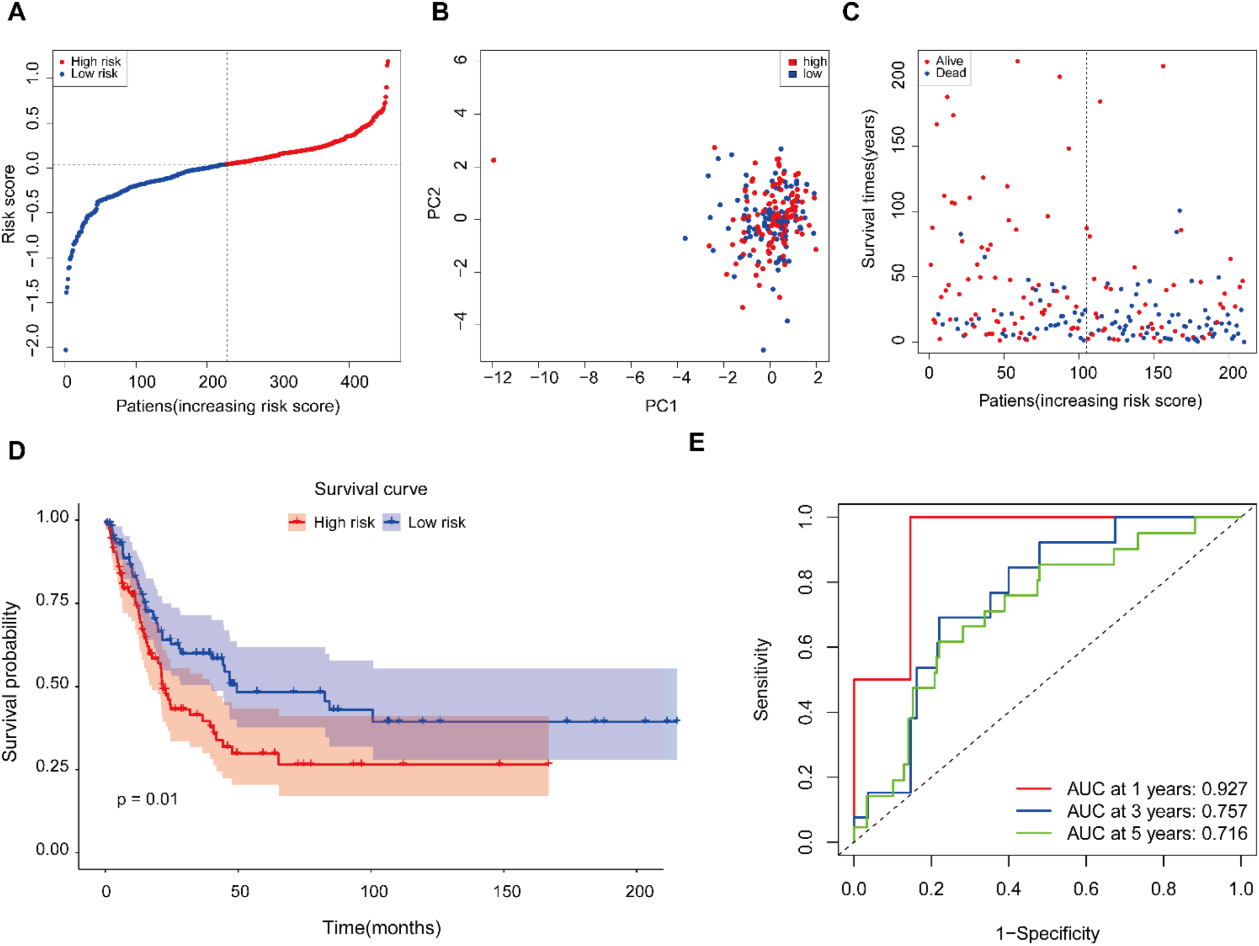
Validation of the ac4C-based prognostic signature in the GEO cohort. **(A)** Distribution of patient risk scores using the TCGA-derived median cutoff. **(B)** Principal component analysis (PCA) illustrating sample separation by risk group. **(C)** Survival status distribution of patients (dotted line indicates risk group division). **(D)** Kaplan–Meier curves demonstrating overall survival differences between risk categories. **(E)** Time-dependent ROC curves assessing prediction accuracy.

To independently validate the prognostic signature, we applied the seven-gene AGS to the external GSE65904 cohort (n = 214). Employing the median risk score from the TCGA cohort as the cutoff defined distinct high- and low-risk patient groups (**Figure 5A**). Principal component analysis showed clear separation between these subgroups (**Figure 5B**), confirming the signature’s discriminative power. Kaplan-Meier analysis revealed a striking survival divergence, with significantly prolonged survival in low-risk patients (**Figure 5C**), affirming the adverse prognosis associated with high ac4C-related transcription (**Figure 5D**). The model achieved time-dependent AUCs of 0.927 (1-year), 0.757 (3-year), and 0.716 (5-year), demonstrating robust short-to intermediate-term predictive accuracy (**Figure 5E**). Together, these results validate the signature’s ability to stratify melanoma patients into distinct prognostic categories.

### 3.5 Clinical validation of the prognostic risk model

**Figure 6:**
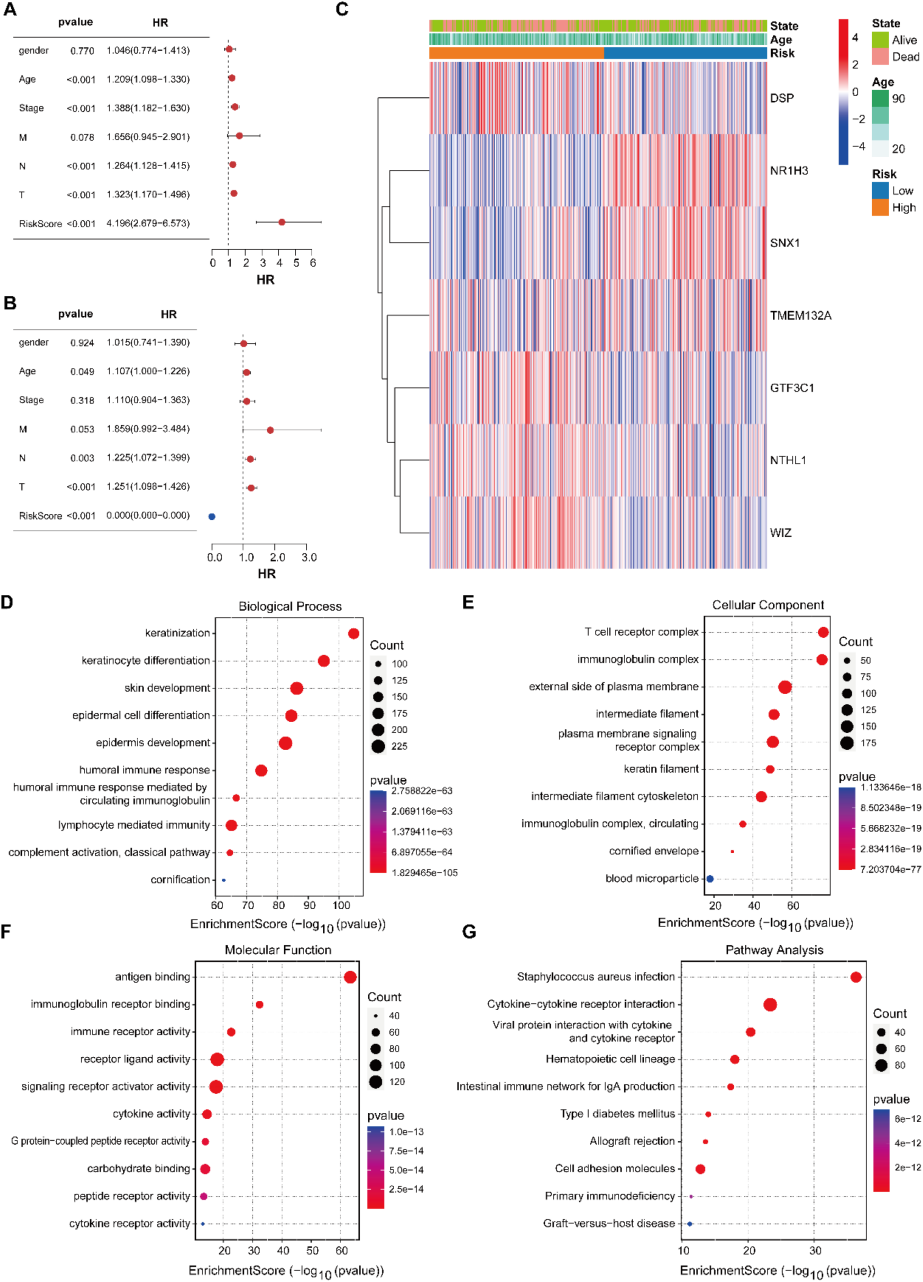
Prognostic independence of the risk score and functional characterization of intergroup DEGs. **(A)** Univariate analysis. **(B)** Multivariate analysis. **(C)** Heatmap visualizing expression patterns of ac4C-related genes across risk subgroups. **(D)** BP cnetplot graph for GO enrichment. **(E)** CC cnetplot graph for GO enrichment. **(F)** MF cnetplot graph for GO enrich-ment. **(G)** Cnetplot graph for KEGG pathways.

Univariate Cox regression identified age, clinical stage, and most notably, the risk score (HR = 4.196, p < 0.001) as significant predictors of overall survival (**Figure 6A**). Multivariate analysis demonstrated that the risk score, along with age and stage, was independently associated with survival (**Figure 6B**). Expression patterns of the seven ac4C-related genes were visualized across risk groups alongside clinical annotations (**Figure 6C**). Notably, DSP, GTF3C1, NTHL1, and WIZ were upregulated in high-risk samples, whereas NR1H3 and SNX1 showed reduced expression, further supporting their involvement in SKCM progression and the biological plausibility of the prognostic signature.

### 3.6 Functional enrichment analysis reveals biological pathways associated with the ac4C Risk model

To investigate pathways associated with the ac4C signature, DEGs (P < 0.05) from TCGA-SKCM were analyzed using GO and KEGG. GO terms were prominently enriched in keratinization, keratinocyte differentiation, epidermal development, humoral immune response, lymphocyte-mediated immunity, and complement activation (**Figure 6D**), highlighting the significance of differentiation and immune modulation in SKCM. Enriched cellular components comprised the T cell receptor complex, immunoglobulin complex, external plasma membrane, intermediate filaments, and cornified envelope (**Figure 6E**), reflecting involvement in immune synapse formation and structural organization. Significant enrichment was observed in molecular functions like antigen binding, immunoglobulin receptor binding, cytokine activity, and peptide receptor binding (**Figure 6F**), highlighting mechanisms associated with immune recognition and intercellular communication. KEGG pathway analysis highlighted enrichment in immune-related and infectious processes, including staphylococcus aureus infection, cytokine signaling, hematopoietic lineage differentiation, mucosal IgA immune network, allograft rejection, and primary immunodeficiency (**Figure 6G**). This reinforces the link between the ac4C risk model and dysregulated immune and inflammatory pathways, suggesting potential for targeted therapeutic strategies.

### 3.7 Characterization of the immune microenvironment reveals distinct phenotypes associated with the AGS

**Figure 7:**
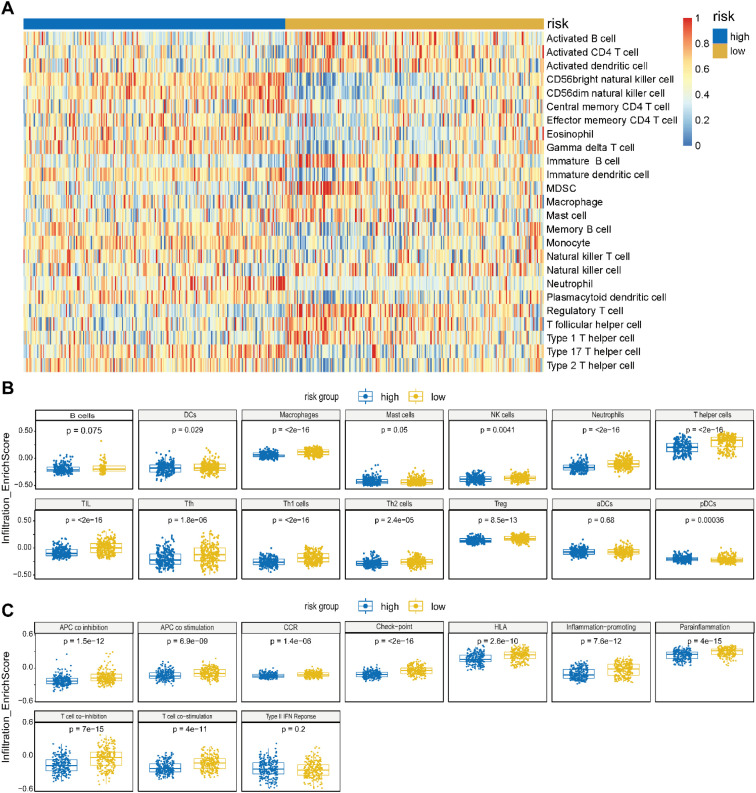
The AGS deciphers the SKCM immune landscape. **(A)** Heatmap depicting immune response profiles between high- and low-risk groups defined by ac4C-related prognostic genes in SKCM. **(B, C)** Enrichment scores of 14 immune cell types **(B)** and 10 immune-related pathways **(C)** in low-risk (yellow) versus high-risk (blue) groups from the TCGA cohort. The ac4C risk model effectively delineates an “immune-active zone” in the low-risk subgroup, contrasting with an “immune desert” phenotype in high-risk patients.

To investigate immune landscape differences underlying the prognostic stratification, we performed ssGSEA - based immune infiltration profiling between the high- and low-risk groups. Across the 28 immune cell populations, low-risk tumors exhibited globally elevated infiltration, whereas high-risk tumors showed a broad reduction, forming a distinct “cold” immune phenotype (**Figure 7A**). Among these, Dendritic cells (DCs), macrophages, NK cells, neutrophils, T helper cells, tumor-infiltrating lymphocytes (TIL), T follicular helper (Tfh) cells, Th1 cells, Th2 cells, and regulatory T cells (Tregs) were all significantly increased in the low-risk group (P < 0.05 for all). These alterations reflect enhanced antigen presentation (DCs), strengthened innate immune activity (macrophages, NK cells, neutrophils), and increased adaptive immune engagement (T helper subsets, TIL, Tfh) (**Figure 7B**). The concurrent elevation of both Th1 and Th2 cells suggests a broad activation of CD4^+^ T-cell–mediated immunity, whereas the rise in Tregs likely represents a compensatory immunoregulatory mechanism accompanying heightened immune activation.

Notably, NK-cell infiltration was significantly higher in the low-risk group (p = 0.0041). NK cells are key innate cytotoxic effectors, defined by their capacity for rapid, antigen-independent tumor cell killing via perforin–granzyme release and recognition of stress-induced or “missing-self” signals. Their increased presence enhances the innate cytolytic arm of the anti-tumor response and reinforces the immune-active phenotype of low-risk tumors.

Consistent with these cellular patterns, most immune functional pathways were significantly upregulated in the low-risk group (**Figure 7C**). Antigen-presentation - related processes, including APC co-stimulation, APC co-inhibition, and HLA expression, were markedly enhanced, indicating strengthened antigen-presenting capacity. Chemokine receptor signaling (CCR), immune checkpoint activity, inflammation-promoting pathways, parainflammation, and T-cell co-stimulation/co-inhibition were also significantly elevated (P < 0.05). In contrast, type II interferon responses showed no significant difference between groups. Together, these findings demonstrate that the low-risk group is characterized by a highly active “immune-hot” microenvironment, whereas high-risk tumors exhibit broad immune suppression, consistent with the observed survival differences.

### 3.8 Evaluation of immune checkpoint expression and associations

**Figure 8:**
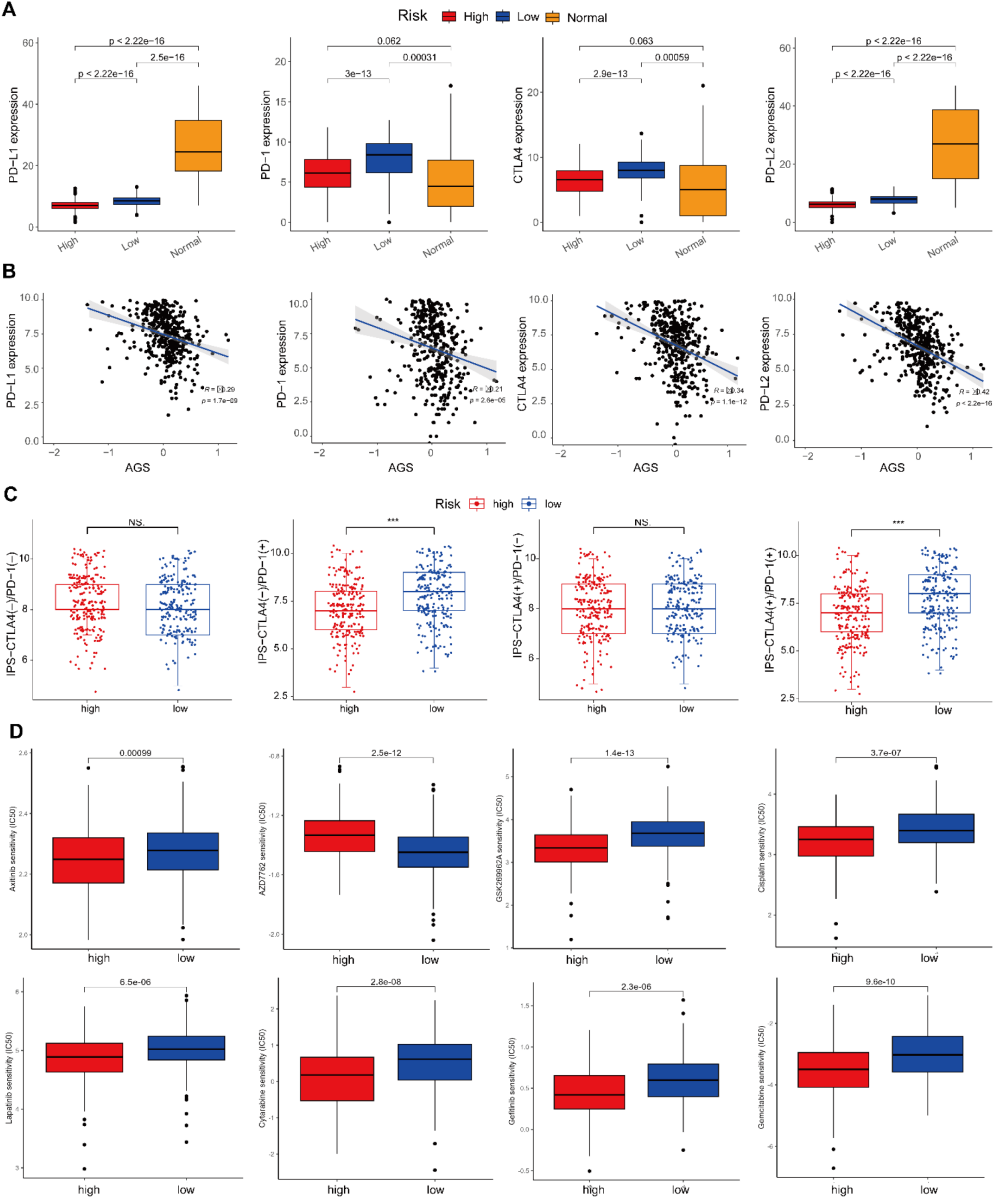
Correlation between AGS and immune checkpoints. **(A)** Immune checkpoint expression profiles stratified by risk group. **(B)** Correlation analysis between the AGS and key immune checkpoints. **(C)** Comparison of IPS between risk subgroups. **(D)** The sensitivity of patients to chemotherapy drugs, like axitinib, AZD7762, cisplatin, lapatinib, cytarabine, gefitinib, gemcitabine, GSK269962A. “*” represents p < 0.05, “**” represents p < 0.01, “***” represents p < 0.001, “ns” represents no significant.

Four clinically relevant immune checkpoints were differentially expressed between the AGS-defined risk groups. Expression of all four checkpoints was significantly lower in the high-risk group compared to the low-risk group (P < 0.001, **Figure 8A**), which is a feature commonly associated with an immune-suppressed tumor microenvironment. Consistently, the AGS showed a significant positive correlation with the overall expression level of these checkpoints (P < 0.05, **Figure 8B**). Further computational prediction using the Immunophenoscore (IPS) indicated that high-risk patients had significantly lower IPS values than low-risk patients in the CTLA-4(-)/PD-1(+) subgroup (**Figure 8C**). These in silico analyses collectively associate the low-risk AGS profile with a more immunogenic microenvironment, while the therapeutic implications of these findings require validation in treated patient cohorts.

### 3.9 Analysis of chemotherapeutic drug sensitivity

Chemotherapy remains a first-line treatment for SKCM, though recurrence and treatment failure often arise due to acquired drug resistance. To assess potential differences in therapeutic sensitivity, IC_50_ values for ten widely used chemotherapeutic agents - axitinib, AZD7762, AZD8055, cisplatin, lapatinib, cytarabine, gefitinib, gemcitabine, bortezomib, and GSK269962A - were compared across risk groups. Results indicated reduced sensitivity in the high-risk group to all agents except AZD7762, to which they showed increased susceptibility (**Figure 8D**, **Supplementary Figure 2**). These findings suggest that the AGS is associated with distinct patterns of computational chemosensitivity, identifying it as a candidate biomarker for future studies investigating treatment stratification.

### 3.10 Multi-level validation of the AGS-derived gene signature in SKCM cell lines, tissues, and independent cohorts

**Figure 9:**
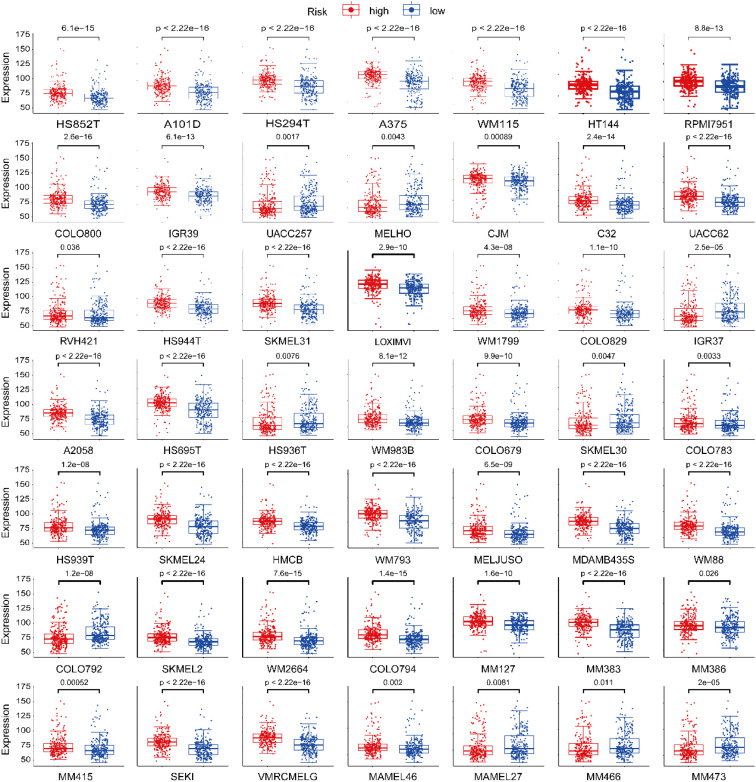
Expression of ac4C-associated genes in SKCM cell lines stratified by risk group.

**Figure 10:**
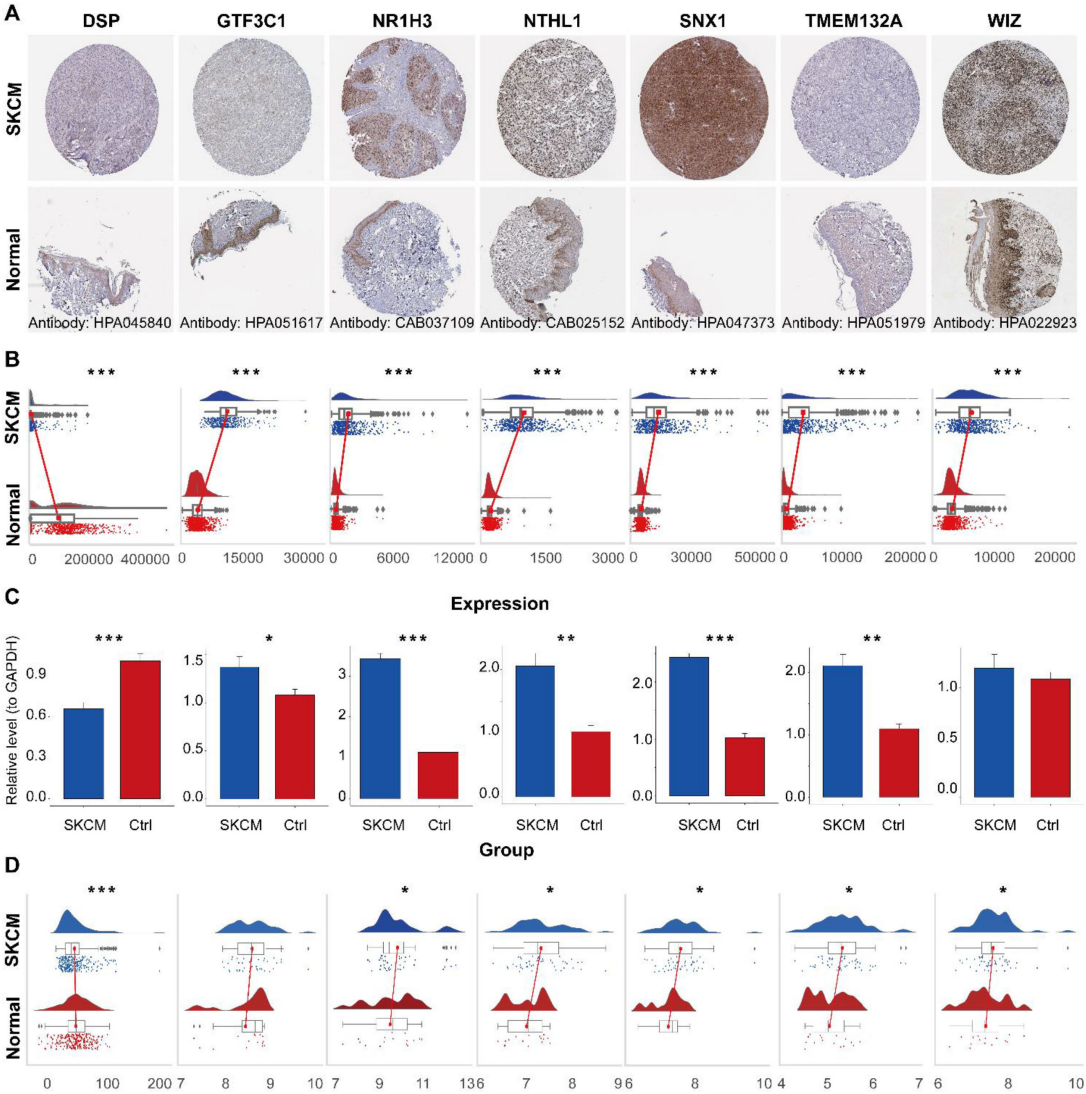
Multi-level validation of the AGS-related gene expression signature in cutaneous melanoma. **(A)** DSP, GTF3C1, NR1H3, NTHL1, SNX1, TMEM132A, WIZ expressions in Skin cutaneous melanoma tissues and normal tissues. **(B)** Expression of the seven prognostic genes in SKCM and normal skin tissues (TCGA cohort). **(C)** Validation of seven genes expression using RT-qPCR (n=5). **(D)** Expression of the seven prognostic genes in SKCM and normal skin tissues (GSE15605). *p < 0.05, **p < 0.01, ***p < 0.001.

For further experimental validation, SKCM cell line expression was examined in relation to AGS -defined risk groups. Analysis of 81 cutaneous-melanoma cell lines revealed distinct transcriptomic profiles between risk-defined subgroups (**Figure 9** and **Supplementary Figure 1**). To obtain protein-level evidence, the expression of signature genes was examined using immunohistochemical images from the Human Protein Atlas. The results showed upregulation of NR1H3, NTHL1, and SNX1, and downregulation of DSP in melanoma compared to normal skin. However, TMEM132A, WIZ, and GTF3C1 showed no statistically significant differential expression (**Figure 10A**). TCGA-SKCM transcriptomic profiling corroborated the differential expression patterns observed, with tumors exhibiting significant mRNA-level deviations from normal tissues (**Figure 10B**).

Experimental validation was conducted to confirm the transcriptional changes suggested by our computational model. We first measured the mRNA expression of the candidate gene NR1H3, NTHL1, SNX, DSP, TMEM132A, WIZ, and GTF3C1 using qPCR in our in-house samples (n=5). Consistent with the bioinformatic prediction, GTF3C1, NR1H3, NTHL1, SNX, TMEM132A and WIZ expressions were markedly upregulated in the tumor group relative to the normal group (**Figure 10C**). Our preliminary qPCR data align with the bioinformatic predictions and are further supported by the independent GSE15605 dataset. Reassuringly, the expression pattern of the genes in this independent cohort mirrored our internal qPCR results, showing a statistically significant difference between the comparable groups (**Figure 10D**). The concordance between our experimental data and public genomic data strengthens the reliability of NR1H3, NTHL1, SNX, DSP, TMEM132A, WIZ, and GTF3C1 as a key molecule associated with the AGS.

## 4 Conclusions and discussion

This study establishes the clinical relevance of a novel seven-gene prognostic signature (AGS) derived from ac4C-associated genes in cutaneous melanoma. Comprising DSP, NR1H3, SNX1, TMEM132A, GTF3C1, NTHL1, and WIZ, the AGS robustly stratifies patients into distinct risk categories and reveals fundamental differences in the tumor-immune microenvironment, distinguishing immunologically active (“hot”) from suppressed (“cold”) phenotypes. The signature further provides clinically relevant and potentially actionable insights, identifying a high-risk subgroup with predicted sensitivity to CHK1/2 inhibition, offering a compelling therapeutic hypothesis for this otherwise difficult-to-treat population. Beyond its prognostic utility, this work underscores the emerging role of ac4C-associated biology in shaping aggressive melanoma phenotypes. While further validation is warranted, the AGS represents a streamlined and biologically informative tool with translational potential.

Collectively, these seven genes form the core components of the ac4C-associated network, functioning as an integrated system within our prognostic model. Their orchestrated interplay enables effective risk stratification in the multivariate model. DSP (25–27) and TMEM132A (28, 29) act as sensors of tissue architecture and oncogenic signaling, jointly activating the Wnt/β-catenin pathway to signal high-risk proliferation. Concurrently, NR1H3 (30–35) and SNX1 (36–38) constitute a regulatory module for the tumor microenvironment and cell death, where their tumor-suppressive and pro-ferroptotic functions collectively report a permissive environment for tumor growth when dysregulated. The cell cycle and transcription are controlled by GTF3C1 (39) and WIZ (40), whose coordinated actions in driving proliferation and stabilizing DNA replication machinery form a key indicator of uncontrolled growth. Completing this system, NTHL1 (41, 42) serves as the genomic fidelity guardian, measuring the tumor’s inherent genomic instability. Thus, by quantitatively integrating signals from these interconnected biological processes - dysregulated signaling, immune and death pathway suppression, uncontrolled proliferation, and DNA damage - our AGS translates complex tumor biology into a precise prognostic bearing for SKCM patients.

Our findings align with the broader understanding of tumor immunology. Both our study and that of Liu et al. indicate that low-risk tumors represent an immunologically active (“hot”) phenotype, characterized by abundant T cells, dendritic cells, and immune checkpoint proteins. This computational profile suggests that low-risk patients might be more likely to respond to immunotherapy. In contrast, high-risk tumors resemble an immunologically ignorant (“cold”) phenotype, with minimal immune infiltration. This, combined with their predicted insensitivity to agents like cisplatin and gemcitabine, points to a particularly challenging clinical subgroup. However, their predicted sensitivity to the CHK1/2 inhibitor AZD7762 suggests a potential dependency on the DNA damage response pathway, offering a rational hypothesis for targeted intervention in high-risk SKCM.

To further contextualize the performance of our ac4C-associated Gene Signature (AGS), we conducted a comparative analysis with several recently published prognostic models in SKCM. The 10-gene ac4C signature (acRGS) developed by Liu et al. achieved 1-, 3-, and 5-year AUCs of 0.67, 0.71, and 0.76 in the training set, with consistent performance in external cohorts (43). Sun et al. constructed a 7-gene cuproptosis-related signature, reporting AUCs of 0.669 (1-year), 0.669 (3-year), and 0.685 (5-year) (44). A lactate metabolism-based model integrating 11 genes and 3 pathological features showed AUCs ranging from 0.61 - 0.78 across two cohorts (45), while a machine learning-derived signature attained 1- to 5-year AUCs between 0.62 - 0.702 in TCGA and up to 0.835 in GEO datasets (46). In comparison, our AGS demonstrated highly competitive, and in some intervals superior, discriminative ability. It achieved 1-, 3-, and 5-year AUCs of 0.785, 0.736, and 0.689 in the TCGA cohort, with robust validation in an independent GEO cohort (0.927, 0.757, and 0.716). Beyond its prognostic performance, the AGS offers distinct advantages. Rooted in the emerging field of ac4C epitranscriptomics, it captures biological pathways distinct from models based on cuproptosis, metabolism, or conventional gene sets. Beyond its prognostic performance, the model also suggests potential therapeutic avenues, including a possible vulnerability to checkpoint kinase - associated pathways.

The AGS model demonstrates promising translational potential by enabling precise risk stratification, and its associated immune and chemosensitivity patterns may ultimately inform individualized surveillance and adjuvant therapy - such as identifying patients more likely to respond to immunotherapy or DNA-damage - response pathway inhibitors. These applications remain speculative and require validation in prospective trials. Although the model has been validated in independent cohorts and supported by RT-qPCR assays, several limitations persist. These include sample heterogeneity, indirect inference of ac4C modification levels, and uneven racial or geographic representation across datasets, all of which must be addressed before robust clinical translation.

An important observation was the absence of significant differential protein expression for three signature genes (TMEM132A, WIZ, and GTF3C1) in the HPA dataset. This finding underscores that the prognostic value of our mRNA-based signature does not depend on binary dysregulation of each gene at the protein level, but rather on their coordinated quantitative transcriptional interplay that reflects an underlying high-risk biological state. This is consistent with the well-documented discordance between mRNA and protein abundance due to post-transcriptional regulation, and suggests that mRNA levels may serve as sensitive indicators of pathway activity - such as Wnt/β-catenin signaling - even in the absence of large changes in steady-state protein expression. Therefore, the model’s strength lies in its capacity to capture a collective transcriptional phenotype. Future proteomic profiling will be necessary to determine how this transcriptomic risk signal maps onto the downstream proteomic landscape.

A central mechanistic question is how the expression of the seven-gene signature reflects the underlying ac4C modification network, given that ac4C primarily enhances mRNA stability and translation efficiency (5, 9). While our study did not directly measure ac4C deposition, we hypothesize that the signature functions as a proxy for the downstream output of dysregulated ac4C-mediated post-transcriptional control. Genes that are direct ac4C targets or regulators may exhibit altered abundance when ac4C activity is perturbed, thereby embedding the epitranscriptomic phenotype within the transcriptome. The most significant limitation is that this framework remains inferential. Future work should employ direct ac4C mapping approaches (e.g., ac4C-seq) to define the modification landscape in SKCM, investigate whether the signature or its associated pathways are enriched for ac4C, and perform functional perturbation of NAT10 to test whether altering ac4C levels modulates the stability, translation, and oncogenic roles of signature genes. Elucidating potential “erasers” and “readers” of ac4C will further clarify the regulatory circuitry captured by the AGS model.

To advance the AGS from a research framework to a clinically mature platform, future work should focus on bridging mechanistic and clinical gaps. This includes functional validation of the key genes, confirmation of predictive performance in retrospective cohorts, and evaluation of potential clinical applications such as risk-stratified adjuvant therapy, immunotherapy guidance for “hot” versus “cold” tumors, and targeted strategies for high-risk patients. Integrating the AGS into a multimodal decision-making framework provides a clear roadmap toward personalized melanoma therapy.

## Data Availability

The original contributions presented in the study are included in the article/**Supplementary Material**. Further inquiries can be directed to the corresponding authors.
