# Supplementary material for: A prognostic signature derived from ac4C-associated genes stratifies survival and tumor immune microenvironment in cutaneous melanoma
Source: Front Immunol. 2026 Jan 7;16:1727135. doi: 10.3389/fimmu.2025.1727135 (PMC12819736; doi:10.3389/fimmu.2025.1727135)
Supplement: Supplementary file 1 [file Image1.pdf]

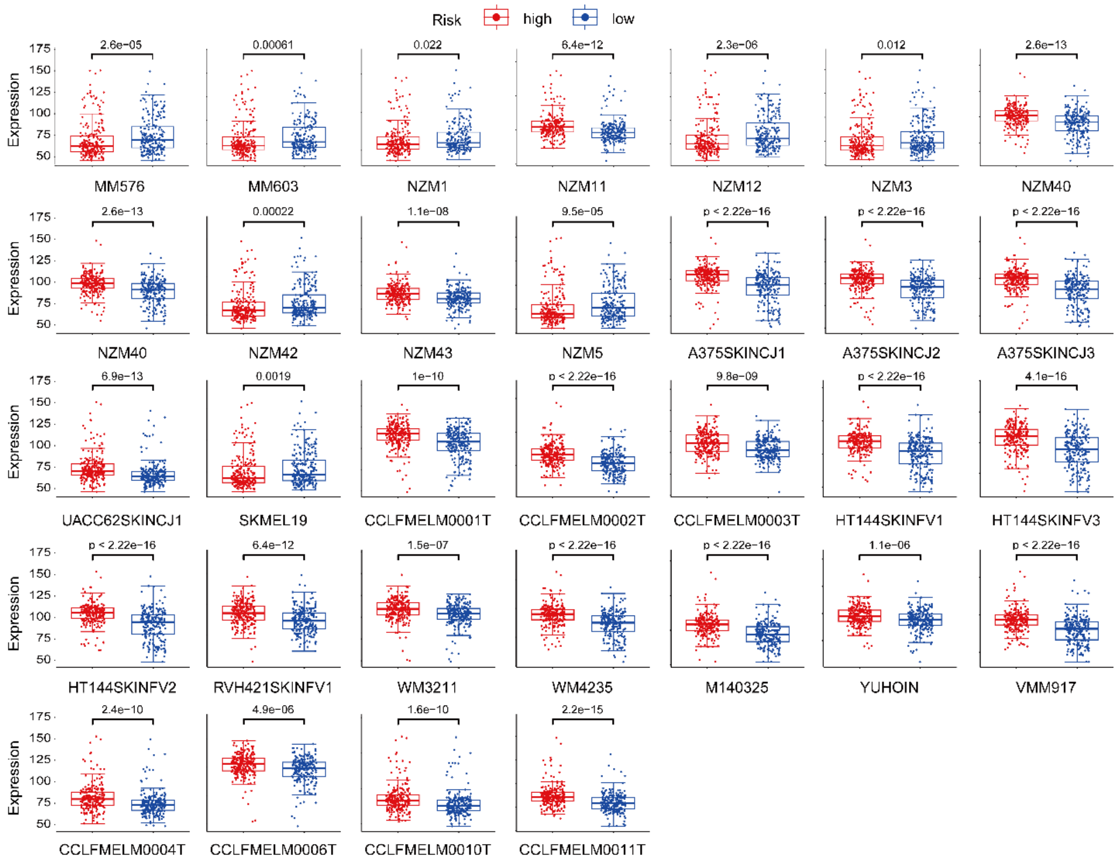

Supplementary Figure 1 Expression of common skin cutaneous melanoma cancer cell lines in high-risk and low-risk groups.

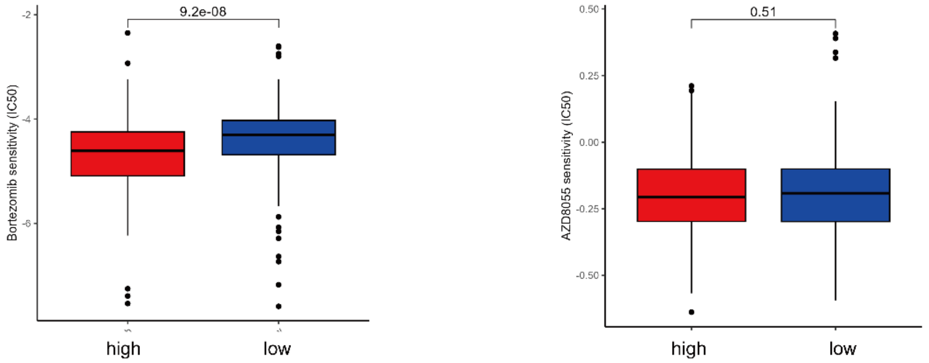

Supplementary Figure 2 The sensitivity of patients to chemotherapy drugs, like AZD8055, bortezomib.

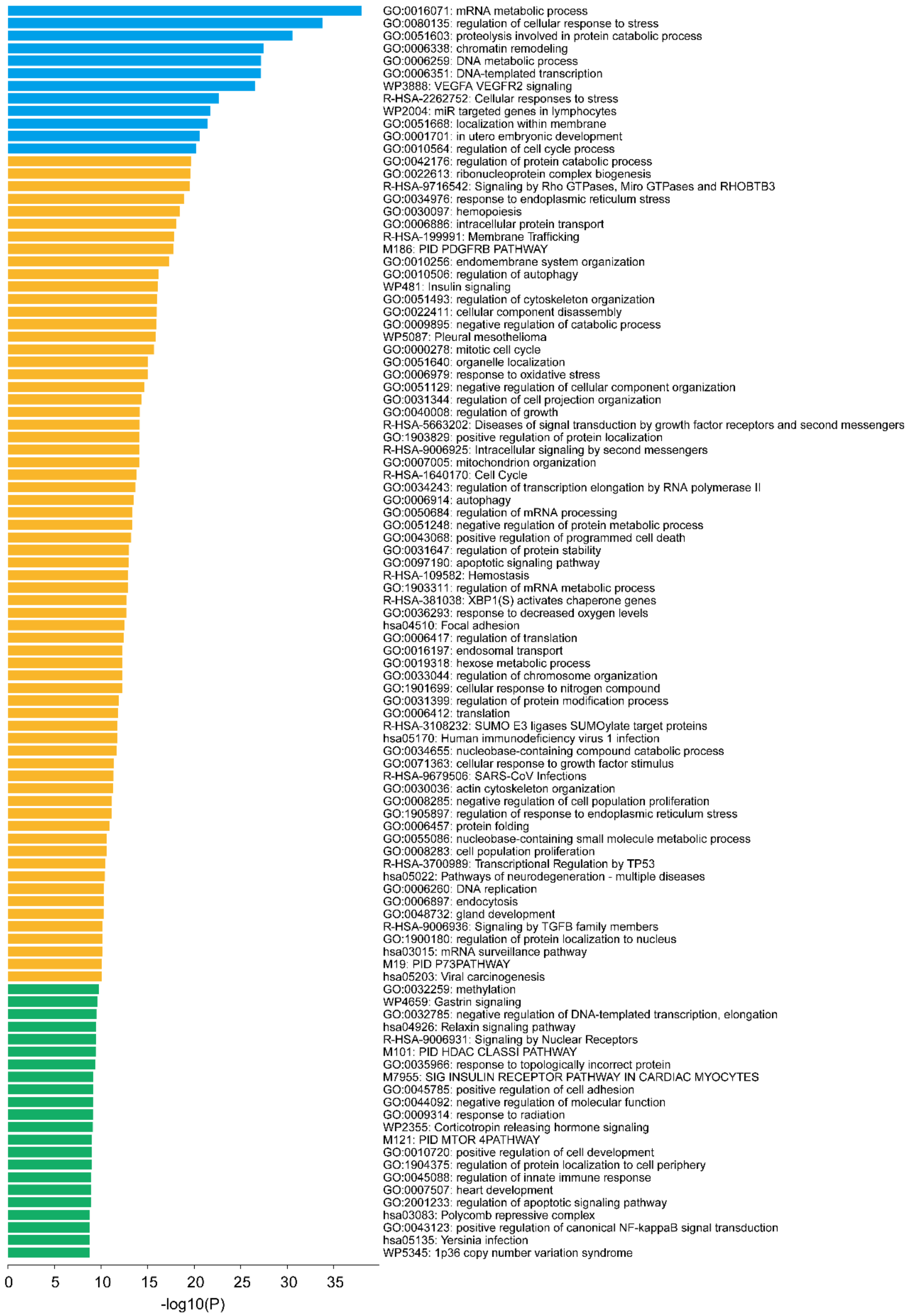

Supplementary Figure 3 Functional Enrichment Analysis of the 2,156 ac4C-Associated Genes. The bar plot displays the top significantly enriched Gene Ontology (GO) terms. The length of the bars represents the -log10(p-value), indicating the statistical significance of the enrichment.
